# Supplementary material for: Role of the visual experience-dependent nascent proteome in neuronal plasticity
Source: eLife. 2018 Feb 7;7:e33420. doi: 10.7554/eLife.33420 (PMC5815848; doi:10.7554/eLife.33420)
Supplement: Supplementary file 5. — Related to Figure 1. Proteins were annotated using PANTHER. The breakdown of the ‘others’ category in the pie charts in Figure 1B–D is included. [file elife-33420-supp5.docx]

**Table S5. List of the PANTHER protein classes in the global brain proteome, nascent proteome, and VE-dependent nascent proteome. Related to Figure 1.**

| PANTHER Protein Class | Percent of gene hits against total # Function hits | | |
| --- | --- | --- | --- |
|  | Global brain proteome | Nascent proteome | VE-dependent nascent proteome |
| nucleic acid binding | 19.0% | 18.7% | 21.6% |
| hydrolase | 9.4% | 9.6% | 11.4% |
| enzyme modulator | 9.2% | 9.6% | 4.5% |
| transferase | 8.6% | 8.7% | 2.3% |
| cytoskeletal protein | 6.5% | 10.3% | 25.0% |
| transcription factor | 6.5% | 5.7% | 9.1% |
| oxidoreductase | 5.8% | 3.7% | 4.5% |
| transporter | 5.0% | 5.3% | 2.3% |
| membrane traffic protein | 3.7% | 3.4% | 1.1% |
| signaling molecule | 3.6% | 2.6% | 1.1% |
| receptor | 3.2% | 2.6% |  |
| ligase | 3.1% | 2.1% | 1.1% |
| chaperone | 1.8% | 3.7% | 4.5% |
| cell adhesion molecule | 1.7% | 2.3% | 3.4% |
| structural protein | 0.8% | 2.0% | 4.5% |
| others | 11.9% | 9.9% | 3.3% |
| Break down for "Others" | | | |
| transfer/carrier protein | 2.80% | 2.10% | 1.10% |
| calcium-binding protein | 2.40% | 2.10% |  |
| isomerase | 1.70% | 1.00% |  |
| extracellular matrix protein | 1.30% | 1.10% |  |
| lyase | 1.30% | 1.30% |  |
| defense/immunity protein | 1.00% | 1.30% |  |
| cell junction protein | 0.70% | 0.50% |  |
| transmembrane receptor regulatory/adaptor protein | 0.40% | 0.40% | 1.10% |
| storage protein | 0.20% | 0.10% | 1.10% |
| surfactant | 0.10% |  |  |
