# Supplementary material for: Role of the visual experience-dependent nascent proteome in neuronal plasticity
Source: eLife. 2018 Feb 7;7:e33420. doi: 10.7554/eLife.33420 (PMC5815848; doi:10.7554/eLife.33420)
Supplement: Supplementary file 6. — Related to Table 1. The synaptic localizations of 83 CPPs are derived from SynProt and PreProt databases. [file elife-33420-supp6.docx]

**Table S6. Summary of synaptic localizations of CCPs. Related to Table 1.**

| Uniprot ID | Gene symbol | SynProt | PreProt | | | |
| --- | --- | --- | --- | --- | --- | --- |
|  |  |  | Active zone | Synaptic Vesicle | Cytomatrix | Active or synaptic vesicle or cytomatrix |
| P68133 | acta1 | V |  | V | V | V |
| P62736 | acta2 | V |  |  |  |  |
| P60709 | actb | V |  | V | V | V |
| P68032 | actc1 | V |  |  |  |  |
| P63261 | actg1 | V |  |  |  |  |
| O94973 | ap2a2 | V |  | V |  | V |
| Q06481 | aplp2 |  |  |  |  |  |
| Q9HBZ2 | arnt2 |  |  |  |  |  |
| O14983 | atp2a1 |  |  |  |  |  |
| P16615 | atp2a2 | V |  | V | V | V |
| Q86VP6 | cand1 | V |  |  |  |  |
| P52907 | capza1 | V |  |  |  |  |
| P47755 | capza2 | V |  | V |  | V |
| P83916 | cbx1 | V |  |  |  |  |
| Q13185 | cbx3 | V |  |  |  |  |
| Q99832 | cct7 | V |  |  |  |  |
| P55287 | cdh11 |  |  |  |  |  |
| Q7Z460 | clasp1 | V |  |  |  |  |
| P39060 | col18a1 |  |  |  |  |  |
| P20674 | cox5a |  | V |  | V | V |
| P35222 | ctnnb1 | V |  |  | V | V |
| Q9H2H0 | cxxc4 |  |  |  |  |  |
| O00429 | dnm1l |  | V |  |  | V |
| Q14195 | dpysl3 | V |  |  |  |  |
| Q14152 | eif3a | V |  |  |  |  |
| P60842 | eif4a1 |  |  |  |  |  |
| P49327 | fasn | V |  | V |  | V |
| P35637 | fus | V |  |  |  |  |
| Q00341 | hdlbp | V |  |  |  |  |
| P62805 | hist1h4a | V |  |  |  |  |
| P19367 | hk1 | V |  | V | V | V |
| Q9UK76 | hn1 |  | V |  |  | V |
| P09651 | hnrnpa1 | V |  | V |  | V |
| Q99729 | hnrnpab |  |  |  |  |  |
| P07910 | hnrnpc | V |  |  |  |  |
| P08238 | hsp90ab1 | V |  |  |  |  |
| P11021 | hspa5 | V |  | V |  | V |
| Q96ST2 | iws1 |  |  |  |  |  |
| A0MZ66 | kiaa1598 |  |  |  |  |  |
| Q12756 | kif1a |  |  |  |  |  |
| O60333 | kif1b |  |  |  | V | V |
| O43896 | kif1c |  |  |  |  |  |
| O60282 | kif5c | V |  |  |  |  |
| P08729 | krt7 |  |  |  |  |  |
| O95678 | krt75 |  |  |  |  |  |
| Q99538 | lgmn |  |  |  |  |  |
| P36776 | lonp1 |  |  |  | V | V |
| P40926 | mdh2 | V |  |  | V | V |
| P50579 | metap2 | V |  |  |  |  |
| Q13765 | naca |  |  |  |  |  |
| P19338 | ncl | V |  |  |  |  |
| Q15233 | nono | V |  |  |  |  |
| Q9H0G5 | nsrp1 |  |  |  |  |  |
| O43252 | papss1 |  |  |  |  |  |
| Q86U86 | pbrm1 |  |  |  |  |  |
| Q15366 | pcbp2 | V |  |  |  |  |
| P57721 | pcbp3 |  |  |  |  |  |
| P02689 | pmp2 |  |  |  |  |  |
| P62333 | psmc6 |  |  |  |  |  |
| Q13200 | psmd2 | V |  | V |  | V |
| P20339 | rab5a | V | V |  |  | V |
| P61020 | rab5b |  |  |  |  |  |
| P51148 | rab5c |  | V |  |  | V |
| P08708 | rps17 | V |  |  |  |  |
| P23246 | sfpq | V |  |  |  |  |
| P42285 | skiv2l2 |  |  |  |  |  |
| Q96GM5 | smarcd1 |  |  |  |  |  |
| Q92925 | smarcd2 |  |  |  |  |  |
| Q6STE5 | smarcd3 | V |  |  |  |  |
| Q13573 | snw1 | V |  |  |  |  |
| Q13813 | sptan1 |  |  |  | V | V |
| Q01082 | sptbn1 | V | V |  |  | V |
| P31948 | stip1 |  | V |  |  | V |
| Q93045 | stmn2 |  |  |  |  |  |
| P08247 | syp | V |  | V |  | V |
| Q9Y490 | tln1 | V |  |  |  |  |
| Q86WT6 | trim69 |  |  |  |  |  |
| Q9BTX7 | ttpal |  |  |  |  |  |
| Q71U36 | tuba1a | V |  | V | V | V |
| P68363 | tuba1b | V |  |  | V | V |
| P08670 | vim | V |  |  |  |  |
| Q14683 | smc1a | V |  |  |  |  |
| P33991 | mcm4 |  |  |  |  |  |
|  | | | | | | |
| Total account gene # | | 45 | 7 | 12 | 12 | 25 |
| % of account gene | | 54% | 8% | 14% | 14% | 30% |
