# Supplementary material for: Role of the visual experience-dependent nascent proteome in neuronal plasticity
Source: eLife. 2018 Feb 7;7:e33420. doi: 10.7554/eLife.33420 (PMC5815848; doi:10.7554/eLife.33420)
Supplement: Supplementary file 7. — Related to Experimental Procedures. Forward and reverse primer sequences that were used for quantification of gene expression are shown. [file elife-33420-supp7.docx]

**Supplementary File S7. RT-PCR primer oligonucleotides used for this study. Related to Experimental Procedures.**

| Gene | Gene name | Primer sequence | | Amplicon size (bp) | Reference |
| --- | --- | --- | --- | --- | --- |
| rps13 | Ribosomal Protein S13 | Forward | ATGTCAAGGAACAGATCTTCAAAC | 131 | Thompson, C.K., and Cline, H.T. (2016). |
|  |  | Reverse | GAGGATTCTCAGGATTTTATTACCA |  |  |
| fus-a | Fused in Sarcoma | Forward | GATACCGCAACATGGCCACCAA | 253 | Dichmann, D.S., and Harland, R.M. (2012). |
|  |  | Reverse | CCAGATCCATACCCTTGTTGT |  |  |
| gria1 | Glutamate Ionotropic Receptor AMPA Type Subunit 1 | Forward | CCCAAATAGCAGCAGACACA | 389 |  |
|  |  | Reverse | CAAGCACTGCCCCTAGAAAG |  |  |
| gria2 | Glutamate Ionotropic Receptor AMPA Type Subunit 2 | Forward | TGAGCAGTGCTGGATAGTGG | 416 |  |
|  |  | Reverse | CAGTAATCAGCCCCCAAAGA |  |  |
